# Phenotypic and Metabolomic Characterization of 3D Lung Cell Cultures Exposed to Airborne Particulate Matter from Three Air Quality Network Stations in Catalonia

**DOI:** 10.3390/toxics10110632

**Published:** 2022-10-22

**Authors:** Paula Villasclaras, Clara Jaén, Barend L. van Drooge, Joan O. Grimalt, Romà Tauler, Carmen Bedia

**Affiliations:** Department of Environmental Chemistry, IDAEA-CSIC, Jordi Girona 18-26, 08034 Barcelona, Spain

**Keywords:** air pollution, particulate matter, sources of pollution, chemometrics, lipidomics, metabolomics, data fusion

## Abstract

Air pollution constitutes an environmental problem that it is known to cause many serious adverse effects on the cardiovascular and respiratory systems. The chemical characterization of particulate matter (PM) is key for a better understanding of the associations between chemistry and toxicological effects. In this work, the chemical composition and biological effects of fifteen PM_10_ air filter samples from three air quality stations in Catalonia with contrasting air quality backgrounds were investigated. Three-dimensional (3D) lung cancer cell cultures were exposed to these sample extracts, and cytotoxicity, reactive oxygen species (ROS) induction, metabolomics, and lipidomics were explored. The factor analysis method Multivariate Curve Resolution–Alternating Least-Squares (MCR-ALS) was employed for an integrated interpretation of the associations between chemical composition and biological effects, which could be related to urban traffic emission, biomass burning smoke, and secondary aerosols. In this pilot study, a novel strategy combining new approach methodologies and chemometrics provided new insights into the biomolecular changes in lung cells associated with different sources of air pollution. This approach can be applied in further research on air pollution toxicity to improve our understanding of the causality between chemistry and its effects.

## 1. Introduction

Air pollution is a major health concern for human populations all over the world. In Europe alone, 307,000 premature deaths are attributed to exposure to particulate matter (PM) [1]. Particles known as PM_10_, with a diameter equal to or smaller than 10 µm, can be inhaled and deposited in the respiratory tract, posing a major risk to human health [2]. Different epidemiological studies indicate that PM is associated with an increasing incidence of respiratory tract diseases, such as asthma, pneumonia, and chronic obstructive pulmonary disease. Exposure to PM is also associated with enhanced incidence of myocardial infarction, stroke, diabetes type II, and lung cancer [3,4].

To assess the health risks due to air pollution exposure, it is necessary to understand the toxicity of the chemicals present in these particles. Although many studies on air pollution toxicity are still carried out using animal models [5,6,7], in recent years, the use of New Approach Methodologies (NAMs) has gained importance in environmental research in an effort to avoid animal testing [8]. NAMs include in vitro assays such as microphysiological systems (cell culture approaches) and high content methods such as omics technologies.

Most of the cell culture approaches involve monolayer cultures of lung primary cells or established cell lines (e.g., A549, BEAS-2B), although more sophisticated cell cultures, such as three-dimensional spheroids [9], air–liquid interface models (ALI) [10] or lung-on-a-chip methods [11], are being progressively introduced to enhance the physiological relevance of the toxicological results.

Omics technologies such as transcriptomics, proteomics, and metabolomics are powerful methods in environmental toxicology. Their use in the development of Adverse Outcome Pathways of toxic compounds has been very useful to describe the key events occurring at different levels of biomolecular organization [12]. Omics has been used to explore biomolecular changes in many exposome studies related to air pollution, using blood derivatives or urine as biological matrices [13,14,15,16]. Regarding the use in microphysiological models, recent studies have employed metabolomics in bronchial cells to study the metabolic disorders induced by different fractions of urban PM [17,18] or benzo[a]pyrene [19], one of the most abundant and toxic components in PM_2.5_ (PM of 2.5 microns or less in diameter). The combination of different omics with toxicological assays provides a holistic view of the cell responses to chemical aggression. For instance, integration of metabolomics, lipidomics, and proteomics analysis has been carried out to study the toxicity of PM_2.5_ in the A549 cell line [20].

A considerable number of research studies focused on PM cytotoxicity do not relate the biomolecular changes observed to specific chemical compounds present in the PM chemical mixture. Due to the dynamic nature of PM, a chemical characterization is needed to understand the toxicological effects produced by certain sources of pollution. In this line, a recent work by Song et al. reported the integration of metabolome, proteome, and cytotoxicity information of BEAS-2B cells exposed to chemically characterized PM_2.5_ from an urban region [21]. In another publication, Xu et al. performed serum metabolomics on rats under controlled exposure to PM_2.5_ with a known composition [22]. For a better interpretation of the chemical mixtures present in PM, its composition can be chemically analyzed and simplified by source apportionment techniques such as Positive Matrix Factorization (PMF) [23] or Multivariate Curve Resolution–Alternating Least Squares (MCR-ALS) [24]. These methodologies have been applied in many air pollution scenarios to decipher the different sources of emission that contribute to the chemical mixtures present in the air [25]. In some of these studies, the source apportionment is accompanied by a toxicological evaluation using A549 cells [26,27,28] or biomolecular artificial mixtures in which the plasmid scission assay to evaluate DNA damage [29] or the dithiothreitol assay to quantify the induction of reactive oxygen species (ROS) [30] are employed. In these cases, the association of the resolved sources of pollution to toxicity, ROS, or DNA damage has been investigated by correlation or linear regression techniques. However, the connections between the air pollution sources and the cellular responses at a biomolecular level are largely unknown.

In this work, fifteen air filter samples from three air quality stations in Catalonia with contrasting air pollution backgrounds were investigated. On the one hand, the source apportionment of chemical data was performed using MCR-ALS. On the other hand, three-dimensional (3D) lung cancer cell cultures were exposed to sample extracts, and lipidomics, metabolomics, cytotoxicity, and reactive oxygen species induction were investigated. Data fusion and further analysis of the chemical and biological profiles using MCR-ALS provided new insights into the toxicity patterns induced by the different sources of pollution.

## 2. Materials and Methods

### 2.1. PM Filters

PM filters were obtained from three different air quality monitoring stations from the Network of Surveillance and Prevention of Air Pollution (XVPCA) of Generalitat de Catalunya in collaboration with the Public Health Agency of Barcelona. The three selected stations were: (1) Barcelona (Eixample), an intensive traffic site located in a densely populated neighborhood; (2) Manlleu, a suburban background site, and (3) Bellver de Cerdanya, a rural background site located in a wide mountain valley in the Pyrenees. A total of fifteen selected PM_10_ filters were used for the present study (five from each station collected on the same five sampling days). All the information about the different locations, selection procedure, and the detailed chemical composition of these PM_10_ filter samples can be found in a previous work [26]. In addition to these filters, one blank filter was used as a reference control in the treatments.

### 2.2. Chemical Reagents

Sodium alginate, sodium chloride, calcium chloride, 2′,7′-Dichlorofluorescin diacetate (H_2_DCFDA), and methionine sulfone were purchased from Merck (Darmstadt, Germany). CellTiter-Blue^®^ viability reagent was purchased from Promega Corporation (Madison, WI, USA). Lipid standards were from Avanti Polar Lipids (Alabaster, AL, USA). Isopropanol was obtained from Carlo Erba Reagents (Sabadell, Spain). Tert-butyl methyl ether (MTBE) was purchased from Scharlab (Barcelona, Spain). High-grade purity methanol and ACN, used as mobile phases, were obtained from Fisher Chemicals (Waltham, MA, USA). Ultrapure water was obtained from the Milli-Q Integral 3 purification system (Merck, Darmstadt, Germany).

### 2.3. Cell Culture

The epithelial lung cell line A549 was obtained from the American Type Culture Collection (ATCC CCL-185) and cultured using Dulbecco’s Modified Eagle’s Medium (DMEM) with Ultraglutamine (BE12-604F/U1, Lonza, Basel, Switzerland) supplemented with 10% of fetal bovine serum (10270-106, Gibco, ThermoFisher, Waltham, MA, USA). Cells were cultured in 75 cm^2^ polystyrene vented flasks, incubated at 37 °C in a humidified incubator set at 5% CO_2_, and passaged every 3–4 days using 0.25% Trypsin-EDTA (Gibco).

### 2.4. PM_10_ Filter Extraction

The filter extracts used to expose A549 cultures were obtained from a 1/8 fraction of each filter (corresponding to 90 m^3^ of sampled air). In addition to the 15 filter samples, 1 blank filter was used and subjected to the same extraction procedure. Each piece of the filter was cut into small pieces and submerged in 4 mL of supplemented DMEM (the same used for cell culture) in a 15 mL plastic tube. Tubes were vortexed for 1 min and then sonicated in a bath for 15 min. The extraction media were then transferred to clean tubes, and 2 mL of supplemented DMEM were added to the filter tube to repeat the procedure and increase the recovery of pollutants from the filter. The liquid phases were then added to the first extraction fractions and were sonicated together for 15 additional minutes to disaggregate the potential particles released from the filter. Tubes were centrifuged at 15,000× *g* rpm for 10 min, and supernatants were recovered in a new tube. These supernatants were sterilized under UV radiation for 1 h before being added to the cell cultures.

### 2.5. Cell Viability Assay

To prepare the viability assays, A549 cells were seeded in flat-bottomed 96-well plates (Nunc) at 0.1 million cells/mL density. After 24 h, cells were attached to the plates and were ready to be treated with the 15 samples and blank extracts. Each well was filled with 100 μL of media. The high exposure dose consisted of 100 μL of the previously obtained extraction media (this dose corresponded to 15 m^3^ of sampled air). The other six decreasing concentrations were prepared by sequential 1:2 dilutions from the first one. Each condition was assayed in triplicate. Culture plates were then incubated for 72 h at standard conditions. After this time, the resazurin cell viability assay was performed using CellTiter-Blue^®^ Reagent following the manufacturer’s indications. After 4 h of additional incubation in the presence of a resazurin reagent, cell plates were read in a fluorescence microplate reader (Infinite M Plex, Tecan, Männedorf, Switzerland) at 560/590 nm excitation/emission wavelengths.

### 2.6. Reactive Oxygen Species (ROS) Assay

Cells were seeded at 0.1 million cells/mL in a flat-bottomed 96-well plate. After 24 h, the medium was aspirated and wells were washed with PBS. Then, 100 μL of an 11 mM glucose solution in PBS containing H_2_DCFDA at 20 μM was added to the wells, and the plate was placed in the incubator under standard conditions for 30 min. Next, wells were aspirated and washed once with PBS before adding 100 μL of PM_10_ extraction samples. The plate was incubated again and the progression of fluorescence intensity was followed for 2 h using a microplate reader (Infinite M Plex, Tecan) at 480/520 nm excitation/emission wavelengths.

### 2.7. Exposure of A549 Alginate Spheroids to PM Extracts

A suspension of cells was mixed in a 1:1 proportion with a 2.4% sodium alginate solution to obtain a final suspension of 7 million cells/mL. The wells of 24-well plates were filled with a 102 mM CaCl_2_ solution. Then, using a 21 G needle coupled to a syringe, the suspension of cells was carefully added, drop by drop, to these wells, where the cell spheroids were formed instantaneously. Next, the CaCl_2_ solution was aspirated and spheroids were washed with 1 mL of 0.9% NaCl solution added to the wells. Then, NaCl was replaced by 1 mL of complete medium, and spheroids were placed in the incubator for 24 h. After this time, the medium was aspirated and replaced by 1 mL of filter extracts at a subtoxic concentration (1.8 m^3^ eq. air/mL). The plate was incubated in these conditions for 72 h. Then, the medium was aspirated and spheroids were washed with PBS before adding 1 mL of lysis buffer (55 mM sodium citrate and 150 mM NaCl) to each well. After some minutes, the spheres were disintegrated and the well content was homogenized with a pipette and transferred to 2 mL Eppendorf tubes under cold conditions. The tubes were centrifuged for 10 min at 10,000× *g* rpm and 4 °C and the pellets were frozen at −80 °C until they were subjected to extraction. This procedure was performed twice to prepare both lipid and metabolite extractions.

### 2.8. Lipid Extraction and LC-MS Analysis

To each tube, 1 mL of a solution of MTBE/MeOH (3:1) and 10 μL of a lipid internal standard mixture (16:0 D31-18:1 phosphatidylethanolamine, (17:1) lysophosphatidylethanolamine, (17:1) lysophosphatidylglycerol, (1, 2, 3, -17:0) triglyceride, 200 pmol/each) were added. Tubes were vortexed and placed in a sonication bath for 15 min. Then, 500 μL of H_2_O/MeOH (3:1) was added to the mixture, and the tubes were vortexed again and centrifugated at 10,000× *g* rpm for 10 min. Next, 300 μL of the upper phase was transferred to new tubes and the solvent was evaporated under N_2_ stream. Dry extracts were reconstituted in 170 μL of ACN, and tubes were centrifuged (10,000× *g* rpm, 5 min); 130 μL was used to prepare the sample glass vials for injection and 20 μL to prepare quality control (QC) samples consisting of a pool of the same volume of all samples.

The LC-MS system consisted of a Waters (Milford, MA, USA) Acquity UPLC system coupled to a Waters LCT Premier orthogonal accelerated time of flight (TOF) mass spectrometer. Ten μL of each sample were injected into the LC system equipped with a Kinetex^®^ C8 reversed-phase column 100 Å (100 mm length × 2.1 mm inner diameter × 1.7 μm particle size, Phenomenex, Torrance, CA, USA). The column temperature was held at 30 °C. The mobile phases were water with 1% 1 M ammonium acetate (NH4Ac), 0.1% acetic acid (phase A), and ACN: isopropanol (7:3) containing 1% 1 M NH4Ac, 0.1% acetic acid (phase B). The gradient separation (flow rate of 400 μL min^−1^) was programmed from 45% A (holding for 1 min) to 35% A for 3 min in a linear gradient, followed by another two linear gradient ramps to 11% A (8 min) and 1% (3 min). Finally, the column was washed for 3 min with 1% A, and the column was re-equilibrated for 4 min in the initial conditions of the chromatographic method (22 min total run time). The TOF mass spectrometer was set to operate both in positive and negative electrospray ionization (ESI) modes. The capillary voltage was set at 3.0 kV for the positive ionization mode, while 2.5 kV was used in the negative mode. In both cases, the desolvation temperature applied was 350 °C, and the desolvation gas flow was set at 600 L h^−1^. Full scan spectra from 50 to 1800 *m*/*z* were acquired, and individual spectra were summed to produce data points every 0.2 s. Leucine was used as an independent reference via the LockSpray interference to maintain mass accuracy and reproducibility of the MS system.

### 2.9. Metabolite Extraction and LC-MS Analysis

To the 2 mL tubes containing the cell pellets, 800 μL of a cold solution of MeOH/H_2_O (8:2) and 10 μL of methionine sulfone as internal standard (200 pmol) were added. Samples were vortexed and placed in a sonication bath for 5 min before centrifugation (10,000× *g* rpm for 5 min). The supernatant was transferred to a new tube and the solvent was evaporated under N_2_ stream. Dry extracts were reconstituted with 170 µL of ACN and centrifuged (10,000× *g* rpm for 5 min). Then, 130 µL was used to prepare the sample glass vials for injection and 20 μL to prepare quality control (QC) samples.

The chromatographic system consisted of a UHPLC instrument (Accela, Thermo Scientific, Waltham, MA, USA) equipped with an Acquity UPLC BEH HILIC column (100 mm length × 2.1 mm inner diameter × 1.7 μm particle size). The temperature of the column oven was set to 50 °C. The mobile phases were ACN/H_2_O (90:10) (phase A) and ACN/H_2_O (50:50), both including 10 mM NH_4_COOH and 0.2% formic acid. The gradient started with 99% A and changed first to 50% in 4 min and then to 10% until minute 6. Then, it returned to 99.9% until minute 6.5 and remained stable until minute 10. An Exactive Orbitrap mass spectrometer (Thermo Scientific) equipped with an HTC PAL autosampler and a Surveyor MS Plus pump was used. The ionization source employed was a heated electrospray (HESI) source operated in the positive mode to obtain MS scans of the precursor ions and all ion fragmentation (AIF) scans where metabolites were fragmented in the HCD collision cell. AIF was performed with a normalized collision energy of 25 eV. Mass spectra were acquired at a resolution of 50,000 FWHM (full width half maximum) at *m*/*z* 200. Working parameters were as follows: electrospray voltage, 3.0 kV; sheath gas flow rate, 45 arbitrary units (a.u.); auxiliary gas flow rate, 10 a.u.; heated capillary temperature, 300 °C; automatic gain control (AGC), 1 × 10^6^; and maximum injection time was set at 250 ms with 2 microscans/scan. The full scan mass range was from *m*/*z* 50 to 1000.

### 2.10. Analysis of LC-MS Data

LC-MS raw data from lipidomics was converted to *cdf* format using Databridge program (Masslynx v4.1, Waters). These data were subjected to the preprocessing and variable selection procedure Regions of Interest (ROI), successfully employed in previous publications dealing with lipidomics and metabolomics data [31,32,33]. In this work, a target list of 112 lipids, very well characterized in our laboratory in terms of *m*/*z* and retention times at the same chromatographic conditions, was analyzed in the LC-MS data. The ROI procedure using this target list produced a matrix of peak areas for each of the lipids investigated in all the analyzed samples. This matrix was further processed by multivariate analysis methods.

In the case of metabolomics data, data were converted from raw to *ibf* format to be analyzed using the MSDIAL program (version 4.9) [34]. File conversion was carried out by the ibfConverter tool of the MSDIAL package. In addition to the variable selection and alignment of features throughout samples, this software package matches AIF-DIA (All ion Fragmentation Data Independent Acquisition) MS2 experimental data, with those stored in public spectral libraries of chemical compounds. This step is necessary to tentatively assign and identify the metabolites present in the analyzed samples. The main set of parameters for MSDIAL analysis is provided in the Supplementary Material. The matrix of peak areas of the detected compounds in all samples was then subjected to multivariate analysis and pattern recognition.

Initial sample exploration of both lipidomics and metabolomics results was carried out by Principal Component Analysis (PCA). Data were autoscaled and PCA models were subjected to cross-validation using the Venetian blinds method. Partial Least Squares–Discriminant Analysis (PLS-DA) was then applied to the same data to understand if samples from different locations and times could be correctly distinguished by their metabolic and lipidomic patterns. The Matthews Correlation Coefficient (MCC) provided information about the method’s sensitivity and specificity. The MCC values varied between +1 (perfect prediction) and −1 (total disagreement between prediction and observation). A value of 0 indicates no better classification than random prediction. In addition, PLS-DA was used to identify the most important lipids and metabolites for the discrimination of classes according to the list of the variables with higher VIP (Variable Importance of the Projection) scores. The variables that had a VIP score >1 in any of the class discrimination models were selected for further lipidomics and metabolomics analyses. For all these variables, fold changes between the mean values of the replicates at each condition and the mean values of the replicates of blank samples were calculated for both lipidomics and metabolomics. As a result, fold change data tables of 15 samples × 70 or 30 variables were obtained for lipidomics and metabolomics, respectively.

### 2.11. Resolution of Chemical, Lipidomic, and Metabolomic Profiles

To investigate the sources of variability of the information gathered in the chemical, lipid, and metabolic datasets and resolve the main profiles of chemical and biological variation, they were processed separately using the Multivariate Curve Resolution–Alternating Least Squares (MCR-ALS) method [24]. This is a bilinear non-negative factor decomposition method based on the following matrix equation, **D = CS^T^ + E,** where the data matrix **D** (I × J) has the chemical, lipid, or metabolic information (j = 1, …J) in all the simultaneously analyzed samples (i = 1, …I). This data matrix is decomposed into the product of two factor matrices, **C** and **S^T^**, which compile the information of the original data matrix **D** in a reduced number of N factors or components (number of columns in **C** and of rows in **S^T^**). The data not explained by the bilinear model are in the residuals data matrix **E**. On the one hand, column vectors of the **C** matrix provide the desired information about the distribution of the N components throughout the samples. On the other hand, row vectors in the **S^T^** matrix give the contribution of the original variables on each of the N components. MCR-ALS is carried out under the constraint of non-negativity to increase the physical interpretability of the resolved components. For each block of data, the selection of the number of components was accomplished considering the total variance explained by the model and the physical interpretability of the different components resolved.

### 2.12. Data Fusion and Analysis

Once the information of the three data blocks (chemistry, lipidomics, and metabolomics) was compressed into MCR-resolved components, the **C** factor matrices obtained in each case were additionally associated with the information from cell viability and ROS production of each analyzed sample. Before this data fusion, variables of each data block were normalized to max–min values between 0 and 1. Then, data were concatenated row-wisely, giving a new data matrix of dimensions 15 × 16, with four types of information combined: chemistry, lipids, metabolites, and phenotype (viability and ROS production). This new data matrix was then again analyzed by MCR-ALS to provide a reduced set of non-negative components which combined the different types of information from the simultaneously analyzed variables. A close analysis of these results enabled the interpretation of the relationship between the PM_10_ samples’ chemistry and their biological effects on lung cells.

### 2.13. Software

All calculations were run on a Fujitsu Celsius R940n workstation equipped with two Intel Xeon CPU E5-2620v3 processors and 128 Gb RAM under Microsoft Windows 7. Data analysis and calculations performed in this work were carried out under a MATLAB R2021a (The MathWorks Inc., Natick, MA, USA) computer environment. PCA and PLS-DA were performed using the PLS-Toolbox (Eigenvector Research Inc., Manson, WA, USA). MS regions of interest (ROI) compression MATLAB routines are described and can be downloaded from [31]. MCR-ALS analysis was performed using the MCR-ALS MATLAB toolbox [35].

The selection procedure for the samples, the chemical composition, and the basic cytotoxicity of the samples employed in the present work were reported in a previous publication [26]. Specifically, samples were from Eixample-Barcelona station (urban traffic site), with heavy traffic in a densely populated neighborhood and high PM_10_ and NO_2_ concentrations; Manlleu (suburban background site), located in an extended plain valley with agricultural fields and livestock farms surrounded by middle-altitude mountains and with one of the highest O_3_ and benzo[a]pyrene concentrations on the Iberian Peninsula and in southern Europe; and Bellver de Cerdanya (rural background site), located in a wide mountain valley in the Pyrenees, surrounded by mountains that are almost 3000 m high and with high O_3_ and benzo[a]pyrene but low NO_2_ concentrations (Table 1). From now on, samples from these air quality stations are called E, M, and B samples, respectively, and were collected on 2 August 2019, 16 November 2019, 20 November 2019, 18 December 2019, and 22 December 2019 (from now on identified with the numbers 1, 2, 3, 4, and 5, respectively). The selection of the sampling days was determined based on their representative air quality indicators profiles, as described in [26].

## 3. Results

### 3.1. Cell Viability and Induction of Reactive Oxygen Species (ROS)

**Figure 1:**
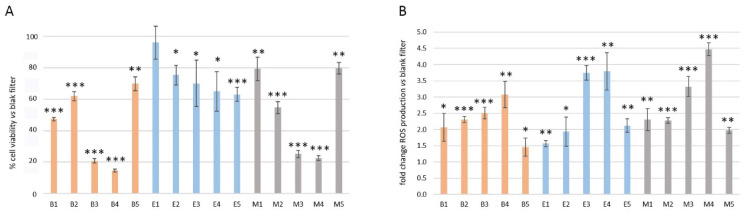
Cytotoxicity and induction of ROS by sample extracts incubated with A549 lung cancer cells. (**A**) Bar plot represents the percentage of cell viability after exposure to each of the sample extracts for 72 hours with respect to the samples exposed to blank samples. (**B**) Bar plot represents the fold change in ROS production after 2 h of cell exposure to each of the sample extracts with respect to cells exposed to blank extracts. Results are representative of *n* = 5 assays (cytotoxicity) and *n* = 3 assays (ROS) performed in triplicate; error bars indicate the standard deviation. Statistical significance was assessed by the T-test. *p* values: * <0.05; ** < 0.01, *** < 0.005.

Figure 1A summarizes the cytotoxicity of the different samples at the maximum concentration tested (15 m^3^ eq. air/mL). The most toxic samples are those from the rural background station B and the suburban station M on the same dates (3 and 4), which induced a decrease of 80% in cell viability, coinciding with stagnant weather conditions. Interestingly, the samples of the urban station E on the same dates presented much less toxicity (around 20–40%), similar to other dates in the same station. The less toxic samples were the ones from summer (samples 1) in the case of E and M stations (10% and 20% of cytotoxicity, respectively) and the samples from the beginning of winter (samples 5), in the case of B and M (30% and 20% of cytotoxicity, respectively), coinciding with advective winds and clean air conditions.

In the present study, a kinetic study of ROS generation under the exposure to PM_10_ filter extracts was performed for 2 h to assess the oxidative potential of samples. The fluorescence reads at 2 h revealed that the most toxic dates (3 and 4) induced the highest levels of oxygenated radicals (from 2.5- to 4.5-fold), this time in all the stations investigated (Figure 1B). These results indicate that there is a relationship between ROS and the induction of cell death that may be common between B and M, probably due to stagnant weather conditions and to a more toxic chemical combination in these stations than that present in the E samples.

### 3.2. Air Quality Parameters and Organic Chemical Composition of Samples

**Figure 2:**
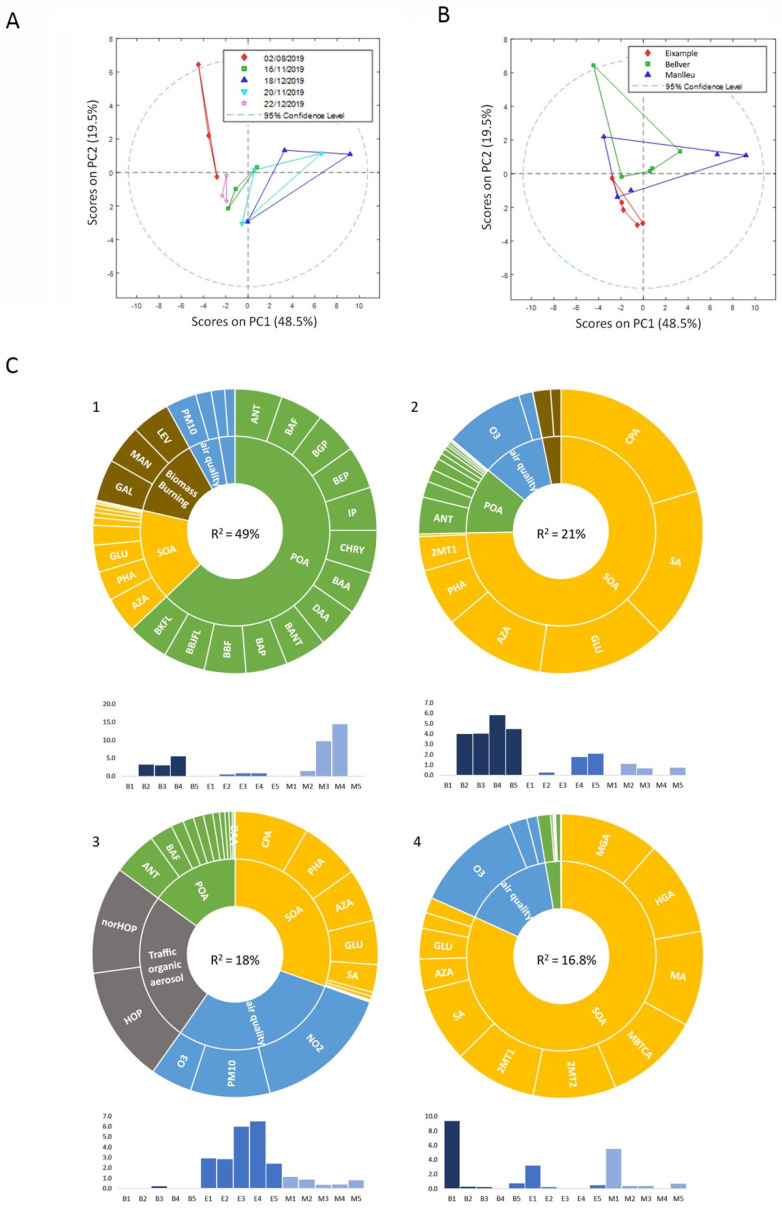
Air quality indicators and chemical analysis of samples. PCA analysis of samples, grouped by (**A**) collection date or (**B**) air quality stations. (**C**) MCR-ALS resolved components of sample chemical information. For each of the components, the solar plot represents their chemical composition, with the compounds classified in different colors according to their chemical nature: air quality indicators, biomass burning organic aerosols, primary organic aerosols (POA), secondary organic aerosols (SOA), and traffic organic aerosols. The bar plots indicate the contribution of each of the samples to the component. Secondary organic aerosols: SA: succinic acid; GLU: glutaric acid; AZA: azealic acid; PHA: phthalic acid; CPA: cis-pinonic acid; MA: malic acid; HGA: 3-hydroxyglutaric acid; MBTCA: 3-methyl-1,2,3-butanetricarboxylic acid; MGA: 2-methylglyceric acid; 2MT1: 2-methylthreitol; 2MT2:2-methylerythriol. Biomass burning aerosols: GAL: galactosan; MAN: mannosan; LEV: levoglucosan. Traffic organic aerosol: norHOP: 17a(H)21β(H)-29-norhopane; HOP: 17a(H)21β(H)-hopane. Primary organic aerosols: BAA: benz[a]anthracene; C + T: chrysene + triphenylene; BBJFL: benzo[b+j]fluoranthene; BKFL: benzo[k]fluoranthene; BEP: benzo[e]pyrene; BAP:benzo[a]pyrene; IP: indeno [123cd]pyrene; DAA: dibenzo[ah]anthracene; BGP: benzo[ghi]perylene; ANT: anthraquinone; BAF: benzo[a]fluorenone; BBF: benzo[b]fluorenone; and BANT: benzantranone. Percentages indicate the variance explained by each component.

Data of the air quality indicators (PM_10_, NO_2_, and O_3_), together with the concentrations of 29 organic tracer compounds previously measured on the 15 samples (see Supplementary Materials), were further investigated for potential source apportionment. Although these tracer compounds only represent a small fraction of the organic aerosol mass, they can be used to successfully reconstruct major emission sources and processes that contribute to air pollution [23]. First, a preliminary analysis of the data by PCA indicated that PC1, which retained 48.5% of the data variance, was able to separate samples by the different dates (Figure 2A), with the dates 3 and 4 being very close to each other and very different from 1 and 5. PC2 (19.5% of data variance) enabled the differentiation between air quality stations, with E and B being the ones that were more different from each other (Figure 2B). To improve the physical interpretation of chemical data and facilitate the association to their biological effects on cells, MCR-ALS was first applied to them to obtain the main chemical source composition profiles, and their distribution within the samples was investigated (Figure 2C). The resolution provided a total of four components that retained 93% of the total variability. As a result, the first component (49% of variance) explained a common trend between B and M samples in 2, 3, and 4 collection dates, being most important for M samples. This component was represented by a high proportion of diverse primary organic aerosols (POA) in a similar proportion, a smaller proportion of secondary organic aerosols (SOA), such as glutaric acid (GLU), azelaic acid (AZA), and phthalic acid (PHA), and a dominant contribution of biomass-burning tracer compounds (mannosan (MAN), galactosan (GAL), and levoglucosan (LEV)) as a result of domestic heating and vegetation waste removal activities carried out in rural and suburban areas on these days in combination with stagnant weather conditions. Component 2 (21% of explained variance) was mainly represented by samples of the rural background station B, and their chemical composition was characterized by SOA, with an important contribution of cis-pinonic acid (CPA), related to the fresh alpha-pinene oxidation, and succinic acid (SA), GLU, AZA, and PHA. Furthermore, smaller fractions of biomass burning tracer compounds, POA, and O_3_ were represented in this component. Component 3 (18% of explained variance) was clearly defined as an urban pollution component, with contributions of tracer traffic aerosols 17a(H)21β(H)-29-norhopane (norHOP) and 17a(H)21β(H)-hopane (HOP), SOA compounds such as CPA, PHA, AZA, and GLU, and the contribution of air quality parameter NO_2_ and PM_10_. Component 4 (17% of explained variance) was represented by biogenic SOA from α-pinene and isoprene oxidation such as 3-methyl-1,2,3-butanetricarboxylic acid (MBTCA), 3-hydroxyglutaric acid (HGA), 2-metylglyceric acid (MGA), 2-methylthreitol (2MT1), and 2-methylerythritol (2MT2). In addition, other SOA species, such as SA and malic acid (MA), as well as O_3_, were represented in this component, which was more abundant in the samples from summer (samples 1), especially in the rural B sample, as could be expected due to higher levels of biogenic emissions from forests in this period of the year.

### 3.3. Lipidomics of Lung Cancer Cells Exposed to PM_10_ Samples Extracts

**Figure 3:**
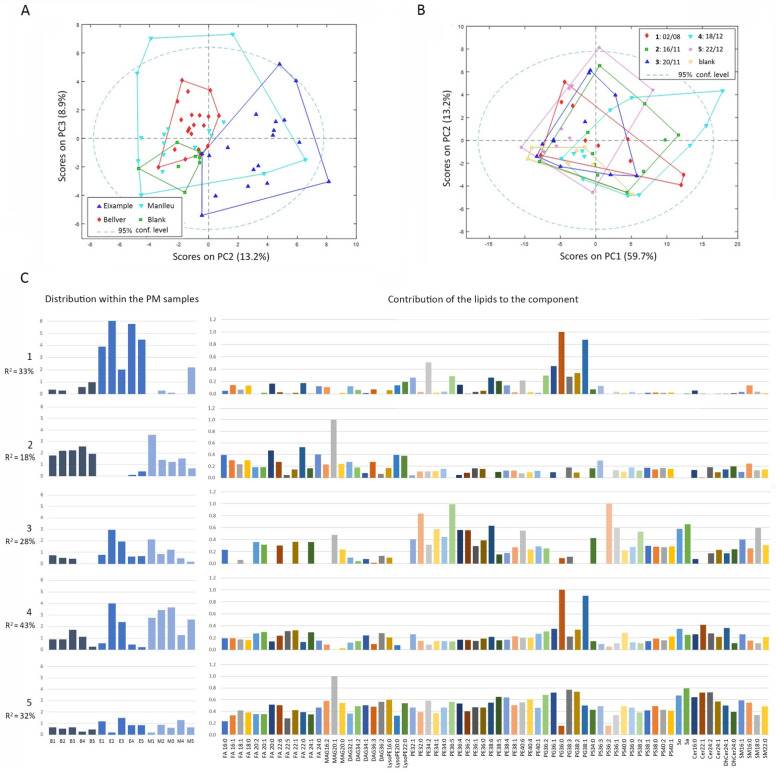
Lipidomics analysis of lung cancer cells exposed to PM_10_ filter extracts. PCA analysis of lipid areas matrix considering (**A**) different locations or (**B**) different collection dates. (**C**) MCR-ALS resolved components from lipidomics data. For each component, the left bar plot represents the sample contribution to the component. The right bar plot indicates the lipid chemical profile of each resolved component. Different color bars in these plots are only meant to facilitate the vertical correspondence to the *x*-axis in all the plots. FA: fatty acid; MAG: monoacylglycerol; DAG: diacylglycerol; PE: phosphatidylethanolamine; PG: phosphatidylglycerol; PS: phosphatidylserine; So: sphingosine; Sa: sphinganine (dihydrosphingosine); Cer: ceramide; DhCer: dihydroceramide; SM: sphingomyelin.

After 72 h of cell incubation with the extracts obtained from the 15 air filters, lipids were extracted and subjected to LC-HRMS. From the LC-HRMS data obtained, the peak areas of 112 lipids (61 and 51, in positive and negative ionization, respectively) were calculated for each of the biological samples and replicates, following a targeted approach (see Methods section). The PCA exploratory analysis of lipidomics data revealed that M samples, due to their high variability, could not be distinguished from the others. However, a 13% variance explained that E samples had a different lipidomic profile from blanks, and 9% of the variance showed differences among B and blank samples (Figure 3A). However, no differences were observed when samples were classified by the collection date (Figure 3B), indicating that the changes in lipid profiles were only location dependent, in this case, only for E and B samples. A PLS-DA model between E and blank samples showed that a cumulative variance of 78% in two latent variables (LV) could discriminate both types of samples, with an MCC = 0.92, indicating that there was very good discrimination between the two groups and that E samples were inducing significant changes in the cell lipid profiles. In the case of the PLS-DA model discriminating between B samples and blanks, 74% of the explained variance from 3 LV was providing perfect discrimination, with an MCC = 1. As expected, samples from M could not be discriminated from blanks. From the successful PLS-DA models, a unique list of lipids was selected using only the features that had VIP values higher than 1 in at least one of the PLS-DA models. As a result, a new data matrix containing the fold changes of 70 lipids in all the samples with respect to the blanks was obtained (see Supplementary Materials). This new fold-change data matrix was further processed by MCR-ALS to obtain the main lipidic profiles found under PM_10_ extracts exposure. As a result, five different components that retained 98.9% of explained variability were resolved (Figure 3C). Component 1 (33% of variance) explained the changes observed in the urban traffic site E samples. The lipidic profile was mainly associated with the increase in two phosphatidylglycerol (PG) species (36:0 and 38:1). Component 2 (18% of variance) was related to B and M samples and was represented by monoacylglycerol (MAG) 20:1 and the increase in four saturated fatty acids (FA) species (16:0, 20:0, 22:0, and 24:0). Component 3 (28% of variance) was common to E2, E3, M1, M2, and M3 and their lipid profile was related to the increase in different phosphatidylethanolamine (PE) and phosphatidylserine (PS) species, sphingosine (So) and sphinganine (Sa), among others. Component 4 (43% of variance) involves the same samples as 3 but with higher contribution, and includes M5, B3, and B4 samples. This component explains again the increase in PG 36:0 and PG 38:1, but this time in association with ceramide (Cer) 22:1 and dihydroceramide (DhCer) 24:1, among other small contributions. Finally, component 5 (32% of explained variance) was found to be present in all the samples, with minor contributions of B4, E2, and M1. The lipid profile contained important contributions of almost all the lipids, with the most intense contribution from MAG 20:1, PGs, and Cer species.

### 3.4. Metabolomics of Lung Cancer Cells Exposed to PM_10_ Samples Extracts

**Figure 4:**
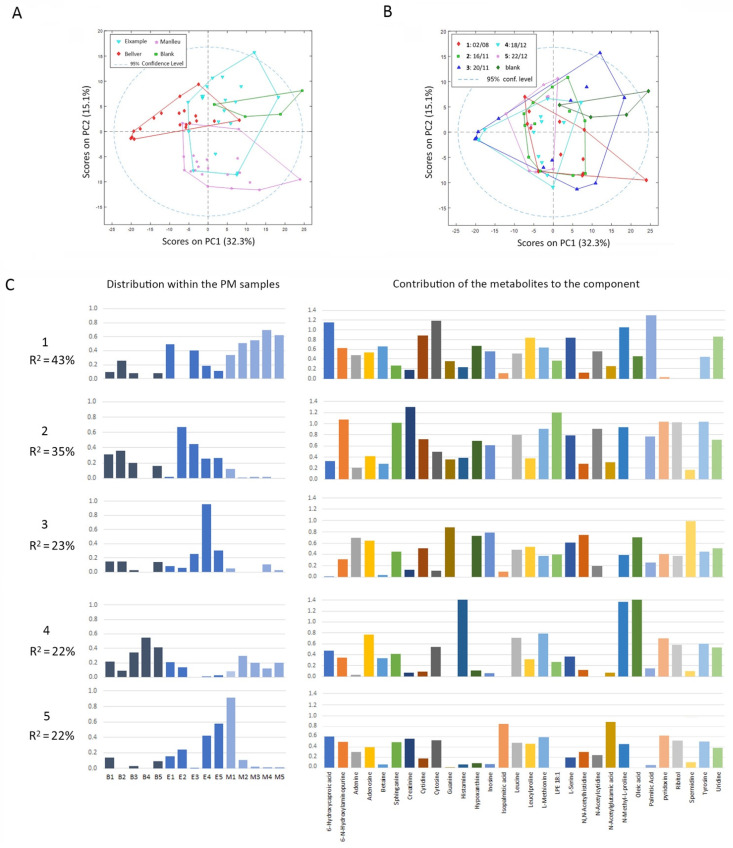
Metabolomics analysis of lung cancer cells exposed to PM_10_ filter extracts. PCA analysis of lipid areas matrix considering (**A**) different locations or (**B**) different collection dates. (**C**) MCR-ALS resolved components of metabolomics data. For each component, the left bar plot represents the sample contribution to the component. The right bar plot indicates the metabolite profile of each resolved component. Different color bars in these plots are only meant to facilitate the vertical correspondence to the *x*-axis in all the plots.

Following a parallel experimental procedure as for lipidomics, metabolites were extracted from A549 lung cancer cells exposed for 72 h to PM_10_ air filter extracts. These metabolite extracts were analyzed using LC-HRMS, and the resulting data were further processed following an untargeted approach by the means of an MSDIAL program. As a result, a matrix of peak areas of 292 features in all the samples was obtained and analyzed by PCA. The PCA scores plot clearly showed differences between B and M samples in respect to blanks and some overlapping between E samples and blanks (Figure 4A). As happened in the lipidomics study, if samples were grouped by their collection date, no time patterns were observed, and they could not be separated in groups (Figure 4B). PLS-DA models were built for each of the air quality station groups in contrast to the blank samples. The PLS-DA model between B and blank samples showed that with one LV (48% of X-variance to explain 64% of Y), sample discrimination was already very good, with an MCC = 0.85. The model between M and blank samples also provided the same discrimination score with one LV (28% of the X-variance to explain 76% of Y). The discrimination between E and blank needed three LV (39% of cumulative X-block variance to explain 93% of Y) to have an MCC = 0.8. Only features identified with an MSDIAL score higher than 80% and that presented a VIP value higher than 1 in any of the PLS-DA models were finally selected for further analysis. As a result, a list of 30 metabolites was obtained, and fold changes between peak areas found in samples exposed to extracts and those exposed to the blank sample were calculated (see Supplementary information). The fold change data matrix was processed by MCR-ALS, resulting in five components that explained 97.5% of the total data variance (Figure 4C). Component 1 (43% of variance) was abundant in the suburban background samples M and also in E1 and E3. In this component, the most relevant compound was palmitic acid, that, together with L-serine, contributes to the initial steps of de novo biosynthesis of sphingolipids (SL). Other important compounds in this component were cytosine, cytidine, and uridine, which are relevant elements of the pyrimidine metabolism. Component 2 (35% of the explained variance) was dedicated to E samples (with the exception of E1) and B samples (except for B4). This was associated with the levels of creatinine, a subproduct of creatine synthesis; lysophosphatidylamine (Lyso PE 18:1); the amino acid tyrosine, a precursor for neurotransmitters and hormones, as well as part of many proteins; pyridoxine; and the sphingolipid (SL) base sphinganine, among others. Component 3 (23% of explained variance) was mostly relevant to sample E4, although it was also found in a small proportion in the other E samples. In this component, spermidine, an aliphatic polyamine involved in many biological processes, and five representative compounds of purine metabolism (guanine, inosine, hypoxanthine, adenine, and adenosine) were found to be very relevant. Component 4 (22% of the total variance) was common to B and M samples, being more abundant in B3, B4, and B5. The most important related metabolites were oleic acid; histamine, a histidine derivative by decarboxylation involved in immunological responses and acting as a neurotransmitter; and N-methyl proline. Finally, component 5 (22% of explained variance) was mostly present in sample M1 and in the majority of E samples. The most relevant metabolites in this component were N-acetylglutamic acid, related to the L-arginine biosynthesis, isopalmitic acid, and pyridoxine, involved in vitamin B6 metabolism.

### 3.5. Joint Analysis of Chemical and Biological Effects Data

**Figure 5:**
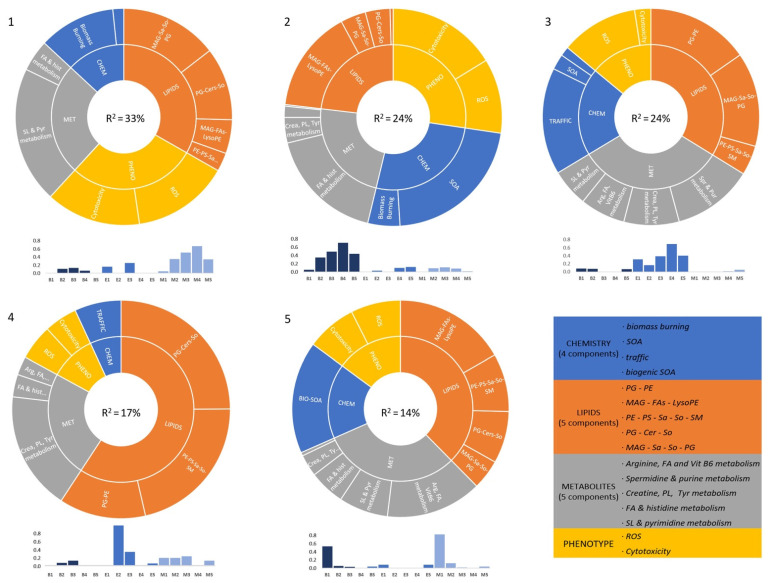
MCR-ALS resolved components of the chemical and biological effects fused data. Graphical representation of each of the MCR-ALS components. Solar plots indicate the contribution of each of the four data blocks on that component. Bar plots indicate the distribution of the component within the samples investigated. Different colors are meant to facilitate the visualization of samples of each air quality station. For more clarity, the composition and the short names of the components previously resolved in each data block are shown on the right-bottom.

The main goal of this work was to investigate how the different chemical profiles defined within samples could be associated with the lipid metabolite of phenotypic patterns obtained as a response of lung cells to the air pollution in sample extracts. With this aim, the C component profiles resulting from the individual MCR-ALS resolutions obtained in the analysis of the three types of variables (chemistry, lipids, and metabolites, in previous sections) can be concatenated in a mid-level data fusion, together with the cytotoxicity and the ROS induction (phenotype matrix) information of the different samples. Therefore, a new 16-column data matrix was built and analyzed by MCR-ALS. As a result, five components representing 91% of the original data were resolved (Figure 5). Three of the components (1, 2, and 3) had a clear distribution on each of the three different air quality stations. In addition, four of the components (1, 2, 3, and 5) have an important contribution of each of the four chemical components previously resolved (biomass burning aerosols, SOA, traffic aerosols, and biogenic SOA). Component 1 (33% of explained variance) associated biomass burning organic aerosols with both cytotoxic effects and the increase in ROS production. This component, mainly distributed in M samples, was also related to the MAG–Sa–So–PG and PG–Cer–So lipid profiles and sphingolipid and pyrimidine metabolism profiles. Component 2 (24% of explained variance) indicated that rural samples of B station, characterized by the presence of SOA and biomass burning aerosols, were associated with the production of ROS and cytotoxicity. Other important contributions were MAG–FA–LysoPE lipid profile and the FA–histidine metabolism profile, previously defined. Component 3 (24% of explained variance), distributed in the urban E samples, was related to the chemical traffic profile, ROS generation, two main lipid profiles (PG–PE and MAG–Sa–So–PG), spermidine and purine metabolism, and creatine–phospholipid–tyrosine metabolism, among other metabolic profiles. Component 4 (17% of explained variance) mainly represented samples E2 and E3 and associated the lipid profiles PG–Cer–So and PE–PS–Sa–So–SM with creatine–phospholipid–tyrosine metabolism with traffic organic aerosols. Component 5 (14% of explained variance) associated the biogenic SOA, mainly present in B1 and M1 summer samples, with some generation of ROS and cytotoxicity, the lipid profile MAG–FAs–LysoPE, and the Arg–FA–vitamin B6 metabolism profile, among other minor contributions.

## 4. Discussion

PM_10_ has been reported as a human health risk factor, but its composition depends on a variety of factors such as meteorological conditions, human and biogenic activities in the sampling area, etc. Therefore, it is necessary to consider PM_10_ as a dynamic chemical mixture when the aim is to investigate the mechanisms of toxicity and health outcomes attributed to its exposure. Bilinear factor analysis methods such as MCR-ALS [24] or positive matrix factorization (PMF) [23] are able to decompose the datasets from complex chemical mixtures to understandable components that can be associated with air pollution emission sources (source apportionment) and atmospheric processing. In this work and the previous publication using the same samples [26], the characterization of important sources of pollution such as traffic emissions, biomass burning smoke, and (biogenic) SOA was possible through the analysis and distribution of specific organic tracer compounds within the factors resolved by MCR-ALS.

The samples selected for the present pilot study were representative of contrasting air pollution scenarios with the intention to illustrate the importance of chemical composition on the diversity of biological effects induced by PM_10_ samples. To add physiological relevance to this omics study, 3D alginate spheroid cultures were employed instead of the traditional adherent cultures. Previous studies have demonstrated that 3D cultures enable the investigation of more realistic interactions between cells and the extracellular media, propitiating a physiological environment closer to the in vivo conditions [36].

Interestingly, at each of the stations investigated, samples of 18/12 (B4, E4, and M4) induced the highest levels of ROS in cells. This is probably related to the high levels of PM_10_ measured for these samples, due to stagnant weather conditions and the presence of Saharan dust [26]. Therefore, one important factor that could have influenced the ROS induction is that sample extracts from 18/12 were more concentrated than on other dates. However, this was not the only possible factor, since other samples with much lower PM_10_ concentration, such as B3 or M3, also presented high levels of ROS. Regarding a potential relationship between ROS induction and cytotoxicity, although ROS was high in all the stations (mostly in 3 and 4 samples), only in B and M stations did these samples cause the highest cytotoxicity, indicating that the different chemical compositions of the three stations are triggering different cellular responses and that ROS does not always involve increased cell death.

Performing a transversal analysis of the different data blocks analyzed is a complicated task, due to the elevated number of variables contributing to each type of information. To simplify the interpretation of the effects related to each of the sources of pollution, the mid-level data fusion of the MCR-resolved components obtained in the analysis of each data block separately, together with the phenotype features (ROS and cytotoxicity), was performed. The MCR-ALS analysis on this new row-wise concatenated/augmented data matrix enabled the finding of connections between different biological effects and, more importantly, finding of potential causality associations between the chemistry (expressed as sources of pollution) and the cell responses. The most relevant results of this data fusion are represented in components 1, 2, 3, and 5, which clearly associated the four sources of pollution resolved (traffic, SOA, biomass burning aerosols, and biogenic SOA, respectively) to different biomolecular and phenotypic changes. In these components, there is a balanced contribution of the four data blocks (sources of pollution, lipids, metabolites, and phenotype), indicating similar importance on them and a high correlation of their elements to characterize the biological effects of the resolved sources of pollution.

In component 1 (33% of explained variance), the suburban background station M, mainly represented by biomass-burning aerosols, was associated with both ROS induction and cytotoxicity. This component showed lipid elements that contained increasing levels of sphingoid bases such as Sa and So and ceramides. The involvement of SL in this component is confirmed by the metabolite block of the component, which had an important contribution of key molecules of the de novo biosynthesis of ceramides, such as L-serine and palmitic acid. Since ceramides are very relevant sphingolipid molecules involved in apoptosis [37], these results suggested that the cell death observed under M samples treatment was probably due to an apoptotic process. This agreed with the decrease in pyrimidine synthesis observed, which is related to the inhibition of proliferation, leading to this type of cell death. In the same line, Pardo et al. have found apoptotic characteristics in lung epithelial cells under biomass burning soluble compounds exposure [38], and de Oliveira Alves et al. found an increase in ROS and apoptotic events in A549 exposed to PM_10_ emitted during biomass burning in the Amazon region [39]. However, this is the first time that the involvement of ceramide has been reported in association with apoptotic cell death under biomass-burning compounds exposure.

In component 2 (24% of explained variance), the rural background samples B were defined by a high proportion of SOA and a fraction of biomass-burning aerosols. These samples involved both cytotoxicity and ROS generation, as in component 1, but the lipid and metabolite profiles associated with these samples indicated different toxicity mechanisms. This component presented a lipid hydrolysis profile containing MAG 20:1 and a variety of FA-saturated species from 16 to 24 carbon length. Additionally, two Lyso PE species containing saturated FA were found to increase. An increase in FA could be a response of cells to obtain energy through β-oxidation as an alternative to glycolysis. In the same line, exposure to acrolein, an environmental pollutant released in ambient air from diesel exhausts and cooking oils, has been found to induce a metabolic shift from the glycolytic pathway to mitochondrial β-oxidation in alveolar type II cells, using palmitate from surfactant phospholipid hydrolysis as an FA source [40]. However, this increase in FA could also be related to the cell death observed. Chu et al. have shown that exogenously added palmitic acid (FA 16:0) increased apoptosis and activation of endoplasmic reticulum (ER) stress mechanisms, such as the unfolded protein response in lung epithelial cells, and that a high-fat diet rich in palmitic acid increased susceptibility to lung fibrosis in mice [41]. In another publication, saturated fatty acids have been found to trigger the ER stress response and induced apoptosis in liver cells via mechanisms that do not involve ceramide accumulation [42].

From the metabolite perspective, a rise in histamine levels was found as a response to the rural background samples. Although the majority of endogenous histamine is found in granules of mastocytes or basophils, less abundant amounts of histamine are distributed in cells from different tissues, such as neurons and the gut. In addition, up-regulation in some cancers of both expression and activity of L-histidine decarboxylase, the enzyme that produces histamine from the amino acid histidine has been found to be associated with increases in cellular proliferation in melanoma, breast, and colorectal cancers [43]. A hypothetical function of histamine as an autocrine growth factor [44] in the context of lung epithelial cancer cells exposed to SOA does not agree with the cytotoxicity observed. However, this proliferative role of histamine could be related to one of the functions of alveolar type II cells, which is to participate in repair processes by initiating recruitment, proliferation, and differentiation into new alveolar epithelial cells [45].

In component 3 (24% of explained variance), which is related to the urban station E, the traffic factor is associated to a lipid profile that involves a high increase in PG species (mainly 36:0 and 38:1, from 4- to 11-fold) and PE 34:2 (from 2- to 5-fold). PG is one of the characteristic phospholipids of pulmonary surfactant in alveolar type II cells [46]. Surfactant is a lipoprotein complex with the essential role of reducing the surface tension at the air–liquid interface of lung epithelia. Surfactant reduces the fluid infiltration into the alveoli and also plays a critical role in lung host defense [47]. A549 cells exhibit features of alveolar type II cells as well as characteristics of bronchial cells. Although the reported content of PG in cultured A549 cells is much lower than that found in primary isolated human type II cells (0.1–0.2% compared to 8% of total phospholipids) [48], the PG levels in A549 are still significantly higher than those found in fibroblasts, indicating that PG plays a role as a surfactant is these cells and that the important increases in PG observed in this component (up to 10-fold) may influence surfactant properties. In this case, an increase in surfactant levels could be interpreted as a protection mechanism of cells to face pollutant aggression, since PG has been shown to block immune system hyper-responsiveness, preserve lung function, and prevent alveolar epithelial injury [49,50].

Traffic pollution is also related to an increase in spermidine levels and a general decrease in purine metabolism. Spermidine, together with putrescine and spermine, belongs to a group of essential organic compounds called polyamines. Polyamines are involved in a wide variety of cellular processes including cell proliferation, cell growth, and inflammation [51]. Spermidine is being increasingly recognized as a potent regulator of inflammatory responses. Specifically in the lungs, it has been found to exert a protective role due to its anti-inflammatory properties [52]. The 1.4- to 3-fold increase in spermidine in E3, E4, and E5 samples could be interpreted as an initial protective reaction of cells to traffic pollution compounds, triggered by ROS production. However, the opposite situation may occur at longer exposures than 72 h, since, if polyamines accumulate excessively within cells or in the extracellular environment, they can be oxidized, generating toxic products and leading to cell death, necrosis, or apoptosis [53].

This protective character of PG and spermidine could explain the lower cytotoxicity of E samples compared to those of M and B under the same exposure time. However, some signs of toxicity leading to cell death also appeared, such as the decrease in purine metabolites. The blockade of nucleotide biosynthesis may be traduced in an arrest of cell proliferation, which may lead to cell death by apoptosis [54]. The reduction in purine synthesis may explain the decrease in creatine metabolism observed, since ATP is necessary to produce phosphocreatine.

Component 4 (17% of explained variance) associated a small proportion of the traffic aerosols from E2 and E3 samples with a small contribution of ROS induction and cytotoxicity. This component was highly represented by lipids. On the one hand, it presented an increase in PG and SLs, but also an increase in certain PE (up to 5-fold) and PS (especially PS 36:2, up to 3-fold). Although in smaller proportions than PG species, PS and PE are also present in the surfactant composition [55]. One possible interpretation of this increase is a cell protection response to more specific urban aerosols exposure, as explained above for PG, in this case involving additional members of the phospholipid family.

Component 5 (14% of explained variance) related the bio-SOA pollution from B1 and M1 summer samples to both ROS and cytotoxicity. Moreover, bio-SOA appeared to be related to a lipid hydrolysis profile with the presence of FA increase, as observed in SOA compounds in component 2, but this time associated with an alteration in arginine and vitamin B6 metabolism. Sphingolipid and pyrimidine metabolism also appeared to be affected, indicating that the cell death observed could be produced by an apoptotic process.

The biomolecular effects of these samples are connected to the different cell death response observed at the higher concentration tested (15 m^3^ eq. air/mL). The results reported in this work showed milder cytotoxicity from urban traffic samples compared to rural and suburban stations under the same exposure time. However, urban samples also showed some lipid and metabolic signatures suggesting incipient cell death. An enhancement in the death of alveolar epithelial cells may interfere with the restoration of the alveolar epithelium, one of the main functions of alveolar type II cells, and, consequently, contribute to the pathogenesis of emphysema, a lung disease characterized by the loss of alveolar cells [56]. The architectural and functional disruptions of the alveolar epithelium in emphysema lead to a reduced gas exchange, which may aggravate other respiratory diseases such as asthma.

At lower concentrations, a different scenario would be expected, in which cell defense mechanisms would be able to overcome the chemical aggressions. However, long-term exposure to these lower concentrations could have unexpected deleterious effects. Epidemiological studies have shown associations between air pollution and an increased risk of lung cancer. In this context, recent research demonstrated that low-level ambient air pollution may induce DNA damage, which is one of the main mechanisms leading to cancer progression [22]. Further research on longer exposures to low levels of air pollution would contribute to understanding the health risk of chronic exposure to specific sources of pollution.

## 5. Conclusions

The present work introduces a new bioanalytical strategy combining new approach methodologies and chemometrics to simultaneously process different types of information (in this case, chemical, omics, and phenotypic information) with the aim of finding causality relationships between air pollution and health outcomes. Four of the components resolved by MCR-ALS represented the main lipid, metabolite, and phenotype associations found in the different background pollution scenarios represented by the samples. This work illustrates the importance of chemical analysis of PM_10_ air samples for toxicology studies, since the chemical profiles present in the samples would trigger different biological pathways in the epithelial alveolar cells A549.

Further studies including larger air filter collections of different air pollution sources will provide more consistent biological information, able to confirm the preliminary results obtained in this pilot study. In addition, long-term experiments using physiologically relevant cell models will be needed to explore potentially toxic effects of chronic exposure. This will represent a better investigation of the causality associations between air chemistry and biological effects and can be useful for risk assessment and risk management policies.

## Figures and Tables

**Table 1:** Information of air quality stations and air quality data.

			Air Quality Data ^2^			
Station	Site Type ^1^	Location	PM_10_ (µg/m^3^)	NO_2_ (µg/m^3^)	O_3_ (µg/m^3^)	Benzo[a]pyrene (ng/m^3^)
Bellver de Cerdanya	Rural Background	42.36828° N; 1.77680° E; 1060 masl	17	7	42	0.7
Eixample (Barcelona)	Urban Traffic	41.38532° N; 2.15380° E; 26 masl	36	49	37	0.2
Manlleu	Suburban Background	42.00331° N; 2.28730° E; 460 masl	36	20	37	1.4

^1^ Site type is based on predominant soil use and emission source. ^2^ Mean values for the five selected sampling days.

## Supplementary Materials

The following supporting information can be downloaded at: https://www.mdpi.com/article/10.3390/toxics10110632/s1, Supplementary information: Chemical composition, fold changes in metabolomics and lipidomics, and MSDIAL parameters.
